# Cost-Utility and Cost-effectiveness of MoodSwings 2.0, an Internet-Based Self-management Program for Bipolar Disorder: Economic Evaluation Alongside a Randomized Controlled Trial

**DOI:** 10.2196/36496

**Published:** 2022-11-01

**Authors:** Mary Lou Chatterton, Yong Yi Lee, Lesley Berk, Mohammadreza Mohebbi, Michael Berk, Trisha Suppes, Sue Lauder, Cathrine Mihalopoulos

**Affiliations:** 1 Institute for Health Transformation Deakin University Geelong Australia; 2 School of Public Health and Preventive Medicine Monash University Melbourne Australia; 3 School of Public Health The University of Queensland Herston Australia; 4 Queensland Centre for Mental Health Research Brisbane Australia; 5 Institute for Mental and Physical Health and Clinical Translation School of Medicine Deakin University Geelong Australia; 6 Biostatistics Unit Faculty of Health Deakin University Geelong Australia; 7 VA Palo Alto Health Care System Palo Alto, CA United States; 8 Department of Psychiatry and Behavioral Sciences School of Medicine Stanford University Stanford, CA United States; 9 Cairnmillar Institute Hawthorn East Australia

**Keywords:** economic evaluation, cost-effectiveness, cost-utility, clinical trial, bipolar disorder, psychoeducation, cognitive behavioral therapy, internet intervention, mania, depression, psychiatry, neuroscience, mental disorders

## Abstract

**Background:**

Internet-delivered psychosocial interventions can overcome barriers to face-to-face psychosocial care, but limited evidence supports their cost-effectiveness for people with bipolar disorders (BDs).

**Objective:**

This study aimed to conduct within-trial cost-effectiveness and cost-utility analyses of an internet-based intervention for people with BD, MoodSwings 2.0, from an Australian health sector perspective.

**Methods:**

MoodSwings 2.0 included an economic evaluation alongside an international, parallel, and individually stratified randomized controlled trial comparing an internet-based discussion forum (control; group 1), a discussion forum plus internet-based psychoeducation (group 2), and a discussion forum plus psychoeducation and cognitive behavioral tools (group 3). The trial enrolled adults (aged 21 to 65 years) with a diagnosis of BD assessed by telephone using a structured clinical interview. Health sector costs included intervention delivery and additional health care resources used by participants over the 12-month trial follow-up. Outcomes included depression symptoms measured by the Montgomery-Åsberg Depression Rating Scale (MADRS; the trial primary outcome) and quality-adjusted life years (QALYs) calculated using the short-form 6-dimension instrument derived from the 12-item version of the short-form health survey. Average incremental cost-effectiveness (cost per MADRS score) and cost-utility (cost per QALY) ratios were calculated using estimated mean differences between intervention and control groups from linear mixed effects models in the base case.

**Results:**

In total, 304 participants were randomized. Average health sector cost was lowest for group 2 (Aus $9431, SD Aus $8540; Aus $1=US $0.7058) compared with the control group (Aus $15,175, SD Aus $17,206) and group 3 (Aus $15,518, SD Aus $30,523), but none was statistically significantly different. The average QALYs were not significantly different among the groups (group 1: 0.627, SD 0.062; group 2: 0.618, SD 0.094; and group 3: 0.622, SD 0.087). The MADRS scores were previously shown to differ significantly between group 2 and the control group at all follow-up time points (*P*<.05). Group 2 was dominant (lower costs and greater effects) compared with the control group for average incremental cost per point decrease in MADRS score over 12 months (95% CI dominated to Aus $331). Average cost per point change in MADRS score for group 3 versus the control group was dominant (95% CI dominant to Aus $22,585). Group 2 was dominant (95% CI Aus $43,000 to dominant) over the control group based on lower average health sector cost and average QALY benefit of 0.012 (95% CI –0.009 to 0.033). Group 3, compared with the control group, had an average incremental cost-effectiveness ratio of dominant (95% CI dominated to Aus $19,978).

**Conclusions:**

Web-based psychoeducation through MoodSwings 2.0 has the potential to be a cost-effective intervention for people with BD. Additional research is needed to understand the lack of effectiveness for the addition of cognitive behavioral tools with the group 3 intervention.

## Introduction

### Background

Bipolar disorder (BD) is a complex mental health condition with multiple and varying states ranging from elevated mood (mania or hypomania) to feelings of hopelessness and sadness (depression) [1]. It consists of several related diagnoses representing a spectrum of illness, including bipolar type I, bipolar type II, cyclothymia, and bipolar not elsewhere classified. The global prevalence of bipolar spectrum disorders is estimated at 0.741% of the adult population, and BD is associated with significant disability and costs to both health care systems and society [2-4].

The primary therapy for BD consists of mood stabilizing medications, including lithium, antipsychotics, and anticonvulsants [5-13]. Psychosocial therapies, including psychoeducation and cognitive behavioral therapy (CBT), are recommended as add-on therapy to medications to reduce relapse through improved medication adherence, identification of early warning signs, self-management, and family communication [14,15]. Psychosocial therapies delivered through traditional face-to-face methods have been shown to be effective and cost-effective adjunctive treatments to pharmacotherapy for people with BD [16-18] and other mental health diagnoses such as anxiety and depression [19].

### Objectives

Internet-delivered psychosocial therapies can overcome several barriers faced when seeking mental health care, such as geographic location, a limited number of service providers, and the cost of treatment. Internet-delivered psychosocial therapies have been shown to be effective and cost-effective for the treatment of depression and anxiety [20,21]. However, the evidence to support the effectiveness and cost-effectiveness of internet-based psychosocial therapies for people with BDs is limited [18,22]. To fill this gap, the MoodSwings 2.0 randomized controlled trial (RCT) was conducted to investigate the efficacy of an internet-based self-guided psychosocial intervention for people with BD [23]. This analysis reports on the within-trial economic evaluation of MoodSwings 2.0 from an Australian health sector perspective.

## Methods

### Overview

This economic evaluation was conducted alongside the RCT of MoodSwings 2.0 (ClinicalTrials.gov NCT02118623 [Australia] and NCT02106078 [United States]) that recruited study participants on the web from anywhere in the world. The RCT was run from 2 study sites located in Geelong, Victoria, Australia, and Palo Alto, California, United States. Details of the study conduct and analysis of the primary study outcomes have been described elsewhere [23].

In brief, adults (aged 21-65 years) with a diagnosis of BD type I, BD type II, or BD not elsewhere classified assessed by telephone using the Structured Clinical Interview for Diagnostic and Statistical Manual of Mental Disorders, Fifth Edition, were eligible. Additional eligibility criteria included access to emergency care, visiting a health care provider at least twice per year for BD treatment, access to the internet and a computer, fluency in English, competence to provide informed consent, and willingness to provide emergency contact details. Once consent was obtained and inclusion and exclusion confirmed, we randomized participants on the secure website to 1 of 3 conditions:

- Group 1: discussion forum only (control)
- Group 2: discussion forum plus psychoeducational modules
- Group 3: discussion forum and psychoeducational modules plus CBT-based interactive tools

Two-step block randomization was used and coded into the website during development. Research staff members were unable to view the randomization code.

For 12 months from randomization, all participants had access to the MoodSwings 2.0 website and their study arm–specific asynchronous peer discussion forum. Moderators screened discussion posts and edited or deleted those with personal contact information, profanity, or distressing content. Group 2 participants were additionally able to access 5 psychoeducational modules delivered biweekly, followed by 4 booster modules delivered at 3, 6, 9, and 12 months. The psychoeducation modules were adapted from a face-to-face clinician-facilitated manualized program, previously evaluated in randomized evaluations [24-26]. The participants randomized to group 3 were able to access the discussion forum, psychoeducation modules, and interactive CBT-based tools. This included development of a life chart, thought monitoring, simple motivational interviewing techniques, self-reflection, problem solving, identification of personal triggers, and a relapse-prevention plan [27].

### Ethics Approval

This analysis was undertaken with data collected as part of the RCT approved by the institutional review board at Stanford University (Stanford, California, United States; project ID 21897) as well as the human research ethics committees at Barwon Health (Geelong; EC00208, project ID 11/73) and Deakin University (Geelong; EC00213, project ID 2021-072). The study was conducted in accordance with the ethical standards of the responsible committees.

### Costs

The recommendations for economic analyses within an international trial suggest that resource use is costed with local unit costs, followed by analysis of heterogeneity [28]. This method requires country-specific unit costs from collaborators. As this study recruited participants on the web from multiple countries, local unit costs were difficult to source. The trial was managed from Australia and the United States, and an Australian health sector perspective was adopted for the economic evaluation. Health sector costs included the costs to deliver the interventions as well as the costs of other health services used by participants during the trial period (refer to Table S1 in Multimedia Appendix 1 [29]).

A microcosting approach was used to estimate the cost to deliver the 3 interventions. We estimated the personnel time required to monitor the internet-based forums as well as time for debriefing with a supervisor. Personnel costs were calculated by multiplying estimated hours by the average wage rate of a research assistant or supervisor, both with 25% added to account for employer overhead costs (eg, space and administrative overheads).

The cost of 2 desktop computers required for research assistants to monitor the internet-based forums was estimated based on an annual lease cost of Aus $800 (Aus $1=US $0.7058) per computer multiplied by the estimated time required to conduct the study (2.24 years).

The development and maintenance cost of the MoodSwings 2.0 website was provided by the study team as a single estimate. This total cost was apportioned across the 3 internet-based interventions based on complexity. The average health sector cost for each intervention group was then calculated based on the number of trial participants.

Information on participant health service use was captured through a self-report resource use questionnaire, the Cornell Service Index [30], at baseline and 3-, 6-, 9-, and 12-month follow-ups. The Cornell Service Index questionnaire asked about the number and types of medical, psychological, acute care, and support services accessed by study participants in the preceding 3 months. Standard Australian unit costs were applied. Intervention costs were added to the 3month health service costs; next, all health care service use costs over the 3- to 12-month follow-ups were summed.

All costs were presented in 2018-19 Australian dollars (Aus $). Discounting was not applied because the study time horizon was 12 months.

### Outcomes

Self-report outcome measures were administered at baseline and 3-, 6-, 9-, and 12-month follow-ups, including the Montgomery-Åsberg Depression Rating Scale (MADRS), Young Mania Rating Scale, and the short-form health survey, 12-item version (SF-12). The MADRS score was a coprimary outcome measure that achieved statistically significant differences among the groups. It was used as an outcome measure for the cost-effectiveness analysis.

The SF-12 was used to measure participants’ health-related quality of life at each assessment time point. A preference-based scoring algorithm using British general population preference weights was applied to calculate utility values at each time point based on 6 questions from the SF-12 (short-form 6-dimension [SF-6D] instrument) [31]. Quality-adjusted life years (QALYs) were then calculated from the SF-6D utility values using the area under the curve method [32]. The use of QALYs in an economic evaluation is also referred to as a cost-utility analysis [33].

### Statistical Analyses

Statistical analyses were conducted using Stata software (version: 17.0; StataCorp LLC). Base case analyses were conducted on an intention-to-treat basis, including all participants with a baseline assessment. Missing cost and utility data were reported using descriptive statistics. The investigation of relationships between complete cost and outcome data with demographic and clinical variables was undertaken using logistic regression analysis.

Costs and utility values were reported at each time point by randomized group using descriptive statistics (mean and SD). The base case analysis used linear mixed effects models to evaluate between-group differences in postbaseline health sector costs, SF-6D utility values, and QALYs. Health sector costs and SF-6D utility values at each follow-up were regressed on time, baseline value, and treatment allocation with adjustment for baseline covariates specified a priori (baseline cost or utility, sex, and national origin). The model accounted for autocorrelation because of repeated measures across follow-ups and used an unstructured covariance matrix that allows all variances and covariances to be distinct.

Incremental cost-effectiveness ratios (ICERs) were calculated as the mean difference in total health sector costs between 2 randomized groups divided by the mean difference in MADRS scores. The 12-month follow-up was considered the primary time point for comparison in the main efficacy analysis, and this time point was adopted for the cost-effectiveness analysis. A nonparametric bootstrap procedure with 1000 iterations was used to calculate CIs around ICERs. Cost-effectiveness planes were constructed by plotting the 1000 bootstrapped incremental costs and incremental MADRS scores.

The incremental cost-utility ratio was calculated by dividing the mean difference in total health sector cost by the mean difference in QALYs. A nonparametric bootstrap procedure with 1000 iterations and the reordered bootstrap percentile method (1000 iterations) was used to estimate 95% CIs around each average incremental cost-utility ratio [34]. An intervention was considered cost-effective if the resulting ICER fell below the generally accepted Australian willingness-to-pay threshold of Aus $50,000 per QALY [35]. The resulting bootstrap iterations were also used to construct cost-effectiveness planes and acceptability curves to represent the uncertainty in the ICER.

Sensitivity analyses were undertaken to test the assumptions regarding missing data, including complete case analysis and multiple imputation for missing data at follow-up [36]. Missing total cost and outcomes data (utility values and MADRS scores) at each time point (3-, 6-, 9-, and 12-month follow-ups) were imputed through a resampling method using single imputation nested in bootstrapping [37]. This method generated a single call to the multiple imputation function in Stata, with chained equations and predictive mean matching, to produce a complete data set. The costs and outcomes were then analyzed with generalized linear models (GLMs) for each bootstrap resample. After the generation of 1000 bootstrap resamples, the reordered bootstrap percentile method was used to estimate 95% CIs around each average ICER [34]. In these sensitivity analyses the mean difference in total health sector costs over the 12-month follow-up among the randomized groups was estimated using GLMs [38] with the gamma family and identity link. The mean difference in QALYs among the randomized groups was estimated using GLMs with inverse gaussian family and identity link. All statistical models were estimated with adjustment for baseline covariates specified a priori (baseline cost or utility, sex, and national origin). The choice of family for each GLM was based on results from modified Park tests [38]. The link for each model was chosen based on a combination of Pearson correlation, Pregibon link, and modified Hosmer-Lemeshow tests [38].

An additional sensitivity analysis was conducted by estimating the intervention cost from population-level rollout. The average cost per study participant for variable cost items (personnel and computers) was added to the average cost of website development and maintenance per potentially eligible Australian with a diagnosis of BD. To provide a conservative estimate of potential users of the MoodSwings 2.0 program, the number of people with BD seeking care was estimated by multiplying the age- and sex-based prevalence of BD by Australian demographic statistics for the population aged 25 to 65 years in June 2018 [2,39]. The estimate was then multiplied by the percentage of people with BD using health care services for their mental health (67.7%) based on an Australian population-based mental health survey [40].

A threshold analysis was also undertaken to estimate the group 2 intervention cost required for the total cost to be the same as group 1 (control).

## Results

### Participant Characteristics

A total of 322 people provided consent and were screened for eligibility, with 304 (94.4%) participants randomized (refer to Figure S1 in Multimedia Appendix 1 [23]). There were no significant differences in baseline characteristics across the randomized groups (Table 1).

Self-reported resource use from the Cornell Service Index questionnaire and quality of life from the SF-12 were completed by 91.4% (278/304) of the participants at baseline, 39.5% (120/304) at 3-month, 33.9% (103/304) at 6-month, 35.5% (108/304) at 9-month, and 29.3% (89/304) at 12-month follow-ups (Table S2 in Multimedia Appendix 1). Overall, of the 304 participants, there were 84 (27.6%) with complete costs and QALYs over the 5 data collection points during the 12-month study period. Comparisons of participants with complete and incomplete data over the entire 12-month period found that sex was the only variable related to incomplete data; however, this may be due to the high percentage of female participants enrolled in the trial (228/278, 82%). It is unlikely that these data were missing not at random, given the similar patterns of missing cost and utility data that were observed across participants; as well as the qualitative differences between missing cost and utility data and their underlying values. On the basis of our exploratory analyses of missing data mechanisms, it was inferred that incomplete cost and utility data were missing at random. Multiple imputation was consequently used to account for missing data, while incorporating sex as a covariate.

**Table 1:** Baseline demographic characteristics of participants randomized to group 1 (control), group 2 (psychoeducation), or group 3 (cognitive behavioral therapy).

		Group 1 (control; n=102)	Group 2 (n=102)	Group 3 (n=100)	Overall sample (N=304)
Age (years), mean (SD)		39.86 (10.62)	38.65 (11.85)	39.93 (11.15)	39.47 (11.19)
Sex, female, n (%)^a^		77 (75.5)	79 (77.5)	72 (72)	228 (82)
**Country, n (%)^a^**					
	United States	41 (40.2)	37 (36.3)	29 (29)	107 (38.5)
	Australia	32 (31.4)	35 (34.3)	26 (26)	93 (33.5)
	Other	23 (22.5)	23 (22.5)	32 (32)	78 (28.1)
**Bipolar type, n (%)**					
	I	50 (49)	62 (60.8)	55 (55)	167 (54.9)
	II	41 (40.2)	36 (35.3)	38 (38)	115 (37.8)
	Not elsewhere classified	11 (10.8)	4 (3.9)	7 (7)	22 (7.2)
**Working, n (%)^a^**		45 (48)	42 (44.7)	48 (55.2)	135 (49.1)
	Full time	26 (27.7)	22 (23.4)	28 (32.2)	76 (27.6)
	Part time	16 (17)	14 (14.9)	12 (13.8)	42 (15.3)
	Casual	3 (3.2)	6 (6.4)	8 (9.2)	17 (6.2)
**Studying, n (%)^a^**		19 (20)	19 (20)	25 (28.7)	63 (22.7)
	Full time	5 (5.3)	2 (2.1)	10 (11.5)	17 (6.1)
	Part time	14 (14.7)	17 (17.9)	15 (17.2)	46 (16.6)

^a^Of the 304 participants, only 278 (91.4%) completed the sex and national origin questions, 275 (90.5%) completed the work status questions, and 277 (91.1%) completed the study status questions.

### Costs

Table 2 details the resources required, unit costs, and total costs for intervention delivery across the randomized groups. The average cost to deliver the control group intervention was estimated at Aus $421 per randomized participant. Group 2 and group 3 delivery costs were estimated at Aus $645 and Aus $714 per randomized participant, respectively.

The average health sector costs at each time point and totaled over the 12-month follow-up are detailed in Table S3 in Multimedia Appendix 1. The average health sector costs were not significantly different among the groups at baseline or over the 4 individual follow-up periods, except for a significant difference between group 2 and group 3 at 6-month follow-up (*P*=.01). The total average health sector cost, including the intervention cost, was lower for group 2 (Aus $9431) than for the control group (Aus $15,175), but this difference was not statistically significant. The total average health sector costs, including the intervention delivery costs, were comparable for group 3 and group 1 (control).

Table S4 in Multimedia Appendix 1 provides the average costs and SDs for participants who completed all Cornell Service Index questionnaires between 3 and 12 months by service use category across the randomized groups. The largest difference among the groups was noted for acute care costs between group 2 (mean Aus $1015, SD Aus $2206) and group 1 (mean Aus $6040, SD Aus $15,152).

**Table 2:** Intervention costs, in Australian dollars (Aus $1=US $0.7058), by randomized group.

Item		Group 1 (control; n=102; forum only)	Group 2 (n=102; forum + psychoeducation)	Group 3 (n=100; forum + psychoeducation + CBT^a^ tools)	Overall sample (N=304)
Website development and maintenance		22,800.00	45,600.00	51,600.00	120,000.00
Desktop computers		1204.59	1204.59	1,180.97	3590.14
**Personnel**					
	Research assistant (monitoring)	14,778.81	14,778.81	14,489.03	44,046.65
	Research assistant (debriefing)	1477.88	1477.88	1448.90	4404.66
	Supervisor (debriefing)	2689.53	2689.53	2636.79	8015.85
Total intervention cost		42,950.80	65,750.80	71,355.69	180,057.29
Average cost per trial participant		421	645	714	592

^a^CBT: cognitive behavioral therapy.

### Health Outcomes

The average MADRS scores were significantly different between group 2 and group 1 (control) at all follow-up time points (*P*≤.05), with a mean difference ranging between 3.6 (95% CI –0.001 to 7.2; 9-month follow-up) and 5.5 points (95% CI 1.8-9.2; 6-month follow-up; Table 3 and Table S5 in Multimedia Appendix 1 [23]). The only significant difference in MADRS scores between group 3 and group 1 (control) was at 6 months with a mean difference of 4.8 points (95% CI 1.0-8.5; *P*=.01).

The average SF-6D utility value was 0.63 at baseline across the randomized groups. From baseline to 12-month follow-up the average QALYs per group were not significantly different, with mean QALYs of 0.627 (SD 0.062) in group 1, 0.618 (SD 0.094) in group 2, and 0.622 (SD 0.087) in group 3 (Table S6 in Multimedia Appendix 1).

**Table 3:** Incremental cost-effectiveness ratios (ICERs), in Australian dollars (Aus $1=US $0.7058), by follow-up period and randomized group.

			Health care costs, mean difference (95% CI)		MADRS^a^ score, mean difference (95% CI)		Cost per point change in MADRS score (ICER)
**Comparison of group 2 vs group 1 (control)**							
	3-month follow-up	–19 (–1677 to 1640)		4 (0.1 to 7.9)		Dominant^b^	
	6-month follow-up	–1300 (–4721 to 2210)		5.5 (1.8 to 9.2)		Dominant	
	9-month follow-up	–879 (–4688 to 2929)		3.6 (–0.001 to 7.2)		Dominant	
	12-month follow-up	–659 (–3488 to 2170)		3.8 (0.01 to 7.6)		Dominant	
	Total 3 to 12 months	–2858 (–10,909 to 5194)		3.8 (0.01 to 7.6)^c^		Dominant (dominated^d^ to 331)	
**Comparison of group 3 vs group 1 (control)**							
	3-month follow-up	113 (–3804 to 4030)		1.1 (–2.8 to 4.9)		103	
	6-month follow-up	–348 (–4639 to 4944)		4.8 (1.0 to 8.5)		Dominant	
	9-month follow-up	–601 (–3957 to 2754)		2.5 (–1.2 to 6.2)		Dominant	
	12-month follow-up	743 (–3957 to 2754)		3.6 (–0.4 to 7.5)		206	
	Total 3 to 12 months	–94 (9422 to 9235)		3.6 (–0.4 to 7.5)^c^		Dominant (dominant to 22,585)	
**Comparison of group 2 vs group 3**							
	3-month follow-up	581 (3747 to 4888)		–1.9 (–6.9 to 3.1)		Dominated	
	6-month follow-up	4339 (940 to 7738)		–1.1 (–5.7 to 3.5)		Dominated	
	9-month follow-up	466 (–2853 to 3784)		0.3 (–5.0 to 4.4)		1553	
	12-month follow-up	2423 (–845 to 5691)		0.7 (–5.7 to 3.5)		3461	
	Total 3 to 12 months	7798 (–2,303 to 17,900)		0.7 (–5.7 to 3.5)^c^		11,140 (dominant to 147)^e^	

^a^MADRS: Montgomery-Åsberg Depression Rating Scale.

^b^Less costly and more effective.

^c^The 12-month follow-up was used because this was the time point prespecified as the primary outcome comparison.

^d^More costly and less effective.

^e^The results are spread across all 4 quadrants of the cost-effectiveness plane, making the CI difficult to interpret.

### Cost-effectiveness and Cost-Utility

**Figure 1:**
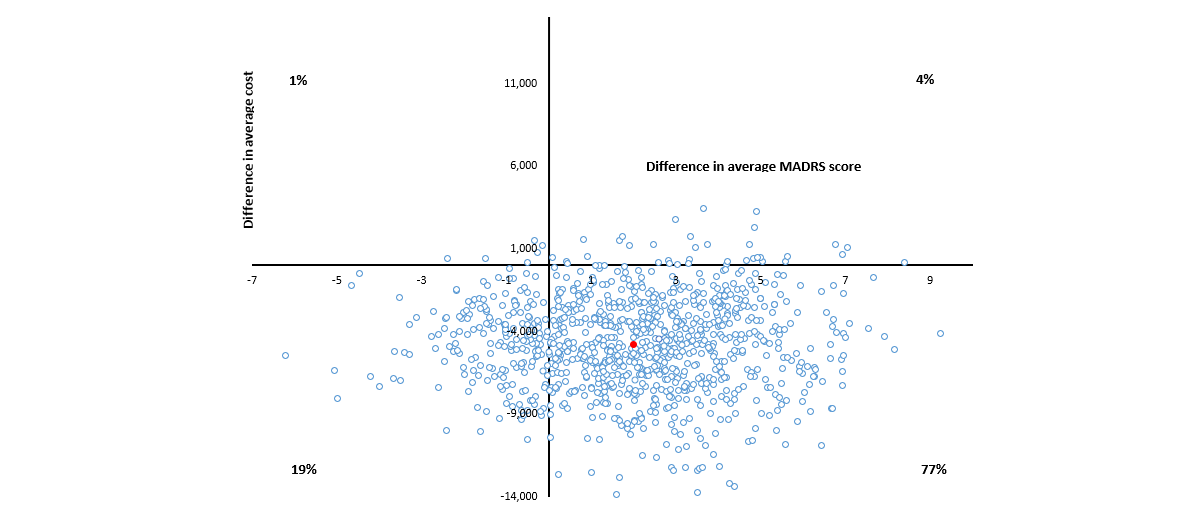
Cost-effectiveness plane, in Australian dollars (Aus $1=US $0.7058), for group 2 versus control cost per Montgomery-Åsberg Depression Rating Scale (MADRS) score improvement bootstrapped from complete cases.

The average incremental cost per point improvement in MADRS scores for group 2 versus group 1 (control) was dominant at each follow-up time point and when summed over the study period (Table 3 and Figure 1). Dominant refers to the scenario when average incremental costs were lower and average incremental effects were higher for the intervention compared with the control group. The 95% CI ranged from dominated (higher incremental cost and negative incremental effect) to Aus $331 per point improvement in MADRS score.

**Figure 2:**
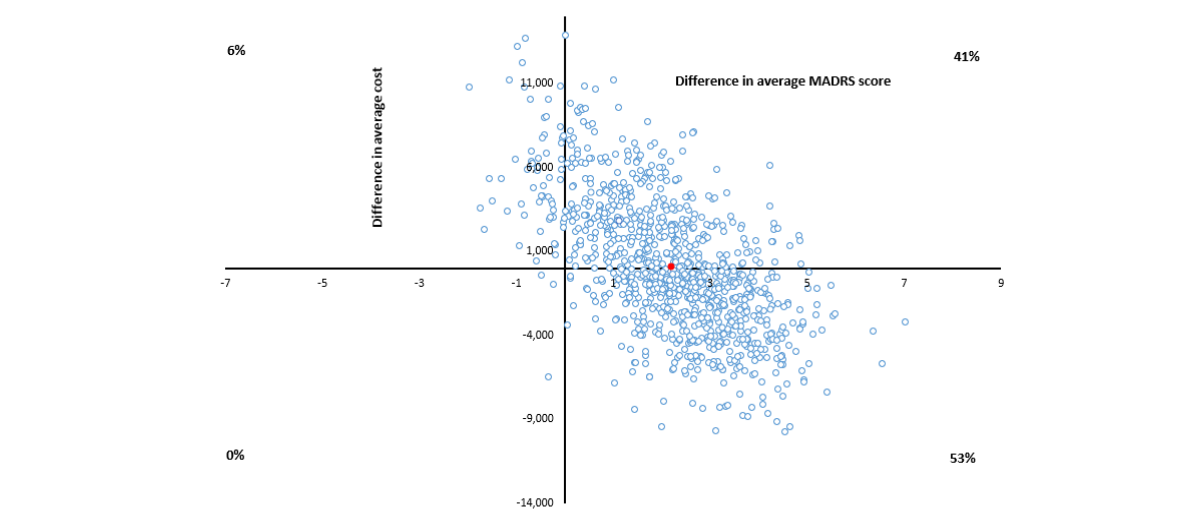
Cost-effectiveness plane, in Australian dollars (Aus $1=US $0.7058), for group 3 versus control cost per Montgomery-Åsberg Depression Rating Scale (MADRS) score improvement bootstrapped from complete cases.

The average costs per point improvement in MADRS score for group 3 versus the control group range from dominant (6- and 9-month follow-ups) to Aus $206 (12-month follow-up). Combining costs over the entire study follow-up leads to the group 3 intervention being dominant (less costly and more effective), with a wide CI from dominant to Aus $22,585 per point improvement in MADRS score (Table 3 and Figure 2).

**Figure 3:**
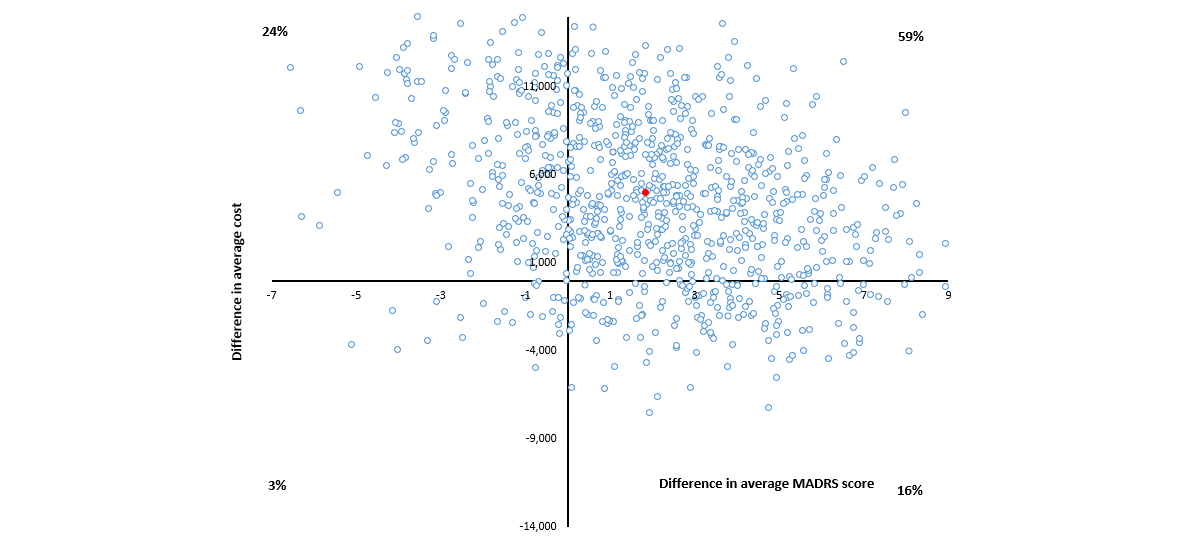
Cost-effectiveness plane, in Australian dollars (Aus $1=US $0.7058), for group 2 versus group 3 cost per Montgomery-Åsberg Depression Rating Scale (MADRS) score improvement bootstrapped from complete cases.

The average costs per point improvement in MADRS score for group 2 versus group 3 range from Aus $1553 (9-month follow-up) to dominated (more costly and less effective at 3- and 6-month follow-ups). Over the entire 12-month period, the average ICER was Aus $11,140 per point change in MADRS score, with a wide spread of bootstrap iterations across all 4 quadrants of the cost-effectiveness plane making it difficult to interpret the CI (Table 3 and Figure 3).

**Figure 4:**
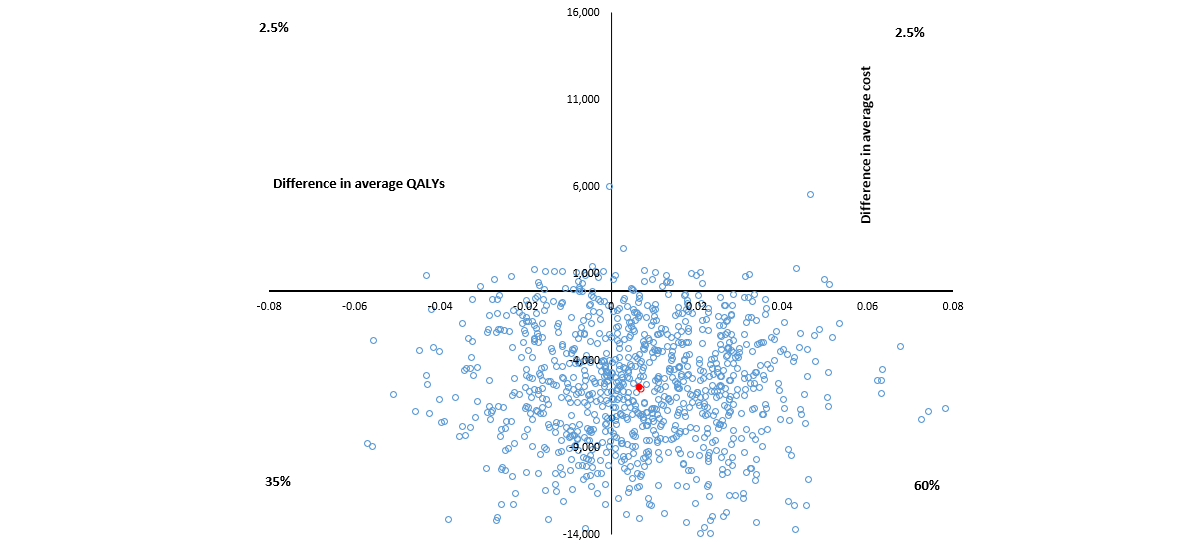
Cost-effectiveness plane, in Australian dollars (Aus $1=US $0.7058), for group 2 versus control cost per quality-adjusted life year (QALY) bootstrapped from complete cases.

The base case cost-utility analysis found that group 2 would be considered the dominant strategy compared with the control group based on the lower average health sector cost and an average QALY benefit of 0.012. The 95% CI for the average incremental cost-utility ratio ranged from Aus $43,000 per QALY to dominant (Table 4 and Figure 4); the lower CI was a result of lower costs and lower incremental QALYs. There was a 79% probability that the psychoeducation modules would be cost-effective at the threshold of Aus $50,000 per QALY.

**Figure 5:**
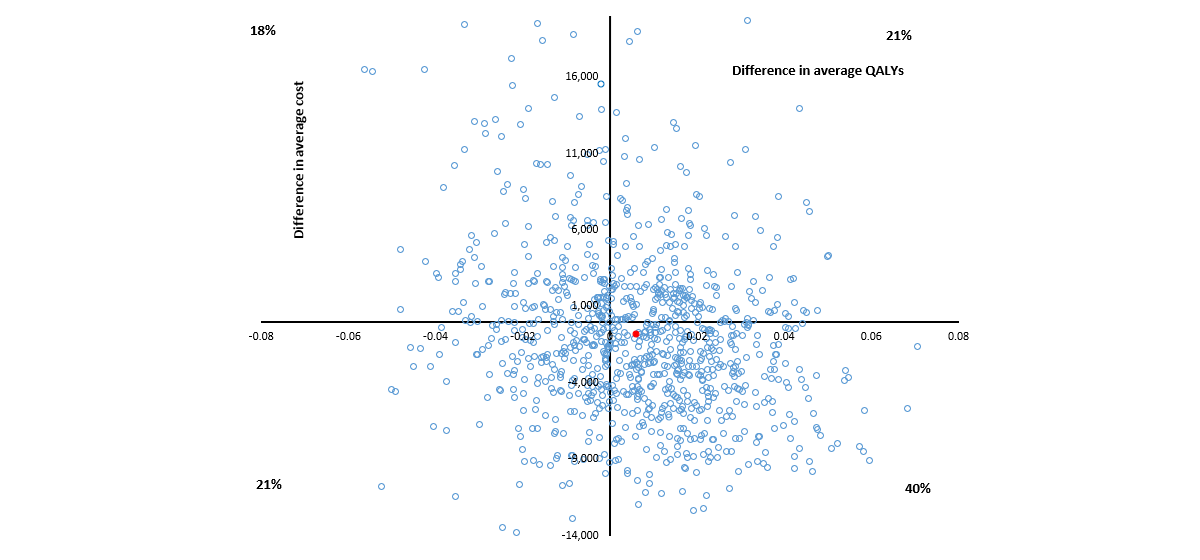
Cost-effectiveness plane, in Australian dollars (Aus $1=US $0.7058), for group 3 versus control cost per quality-adjusted life year (QALY) bootstrapped from complete cases.

The base case average incremental health sector cost for group 3 compared with the control group was estimated at –Aus $94 with an average benefit of 0.002 QALYs resulting in a dominant average ICER (95% CI dominated to –Aus $19,978; Table 4 and Figure 5). The CI is difficult to interpret because the bootstrap iterations span all 4 quadrants on the cost-effectiveness plane. The probability of the combination of psychoeducation and CBT tools being cost-effective at the threshold of Aus $50,000 per QALY was estimated at 51%.

**Figure 6:**
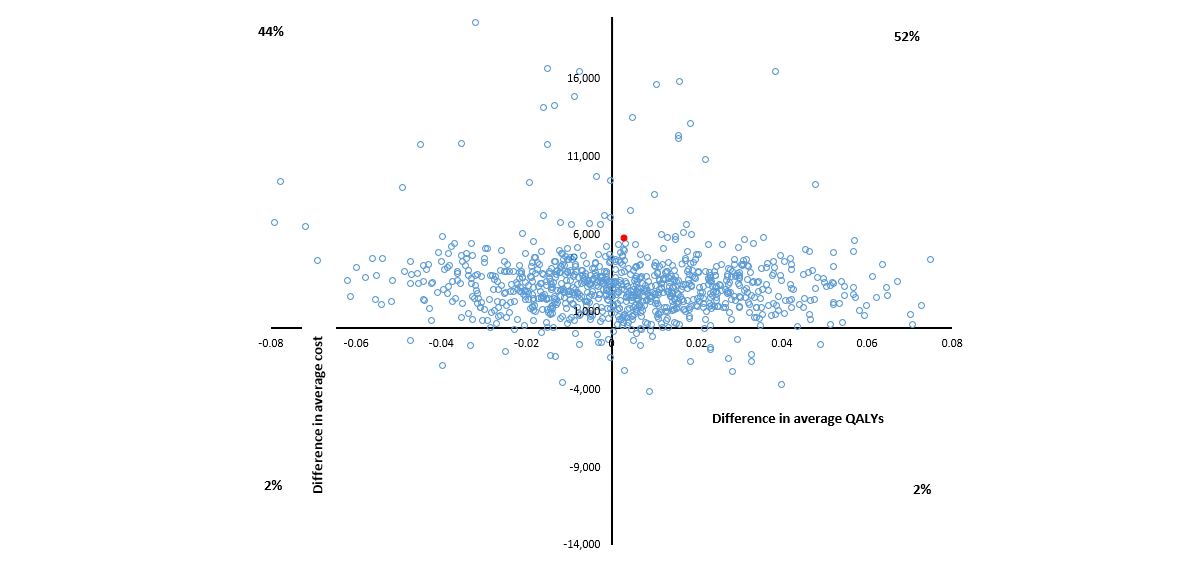
Cost-effectiveness plane, in Australian dollars (Aus $1=US $0.7058), for group 2 versus group 3 cost per quality-adjusted life year (QALY) bootstrapped from complete cases.

Group 3 was dominated by group 2 in the base case because of higher average costs (Aus $7798) and fewer QALYs (–0.004; Table 4 and Figure 6). At the willingness-to-pay threshold of Aus $50,000 per QALY, the probability that the group 3 intervention would be cost-effective compared with the group 2 intervention was estimated at 7%.

**Table 4:** Incremental cost-utility ratios, in Australian dollars (Aus $1=US $0.7058), by follow-up period and randomized group.

			Health care costs, mean difference (95% CI)		Utilities and QALYs^a^, mean difference (95% CI)		Cost per QALY, ICER^b^ (95% CI)
**Comparison of group 2 vs group 1 (control)**							
	3-month follow-up	–19 (–1677 to 1640)		0.0005 (–0.003 to 0.004)		—^c^	
	6-month follow-up	–1300 (–4721 to 2210)		0.003 (–0.003 to 0.010)		—	
	9-month follow-up	–879 (–4688 to 2929)		0.004 (–0.003 to 0.01)		—	
	12-month follow-up	–659 (–3488 to 2170)		0.004 (–0.004 to 0.013)		—	
	Total 3 to 12 months	–2858 (–10,909 to 5194)		0.012 (–0.009 to 0.033)		Dominant (43,000 to dominant)^d^	
**Comparison of group 3 vs group 1 (control)**							
	3-month follow-up	113 (–3804 to 4030)		0.0007 (–0.004 to 0.005)		—	
	6-month follow-up	–348 (–4639 to 4944)		0.002 (–0.007 to 0.010)		—	
	9-month follow-up	–601 (–3957 to 2754)		–0.0005 (–0.008 to 0.007)		—	
	12-month follow-up	743 (–3957 to 2754)		–0.0004 (–0.011 to 0.010)		—	
	Total 3 to 12 months	–94 (–9422 to 9235)		0.002 (–0.023 to 0.027)		Dominant (dominated to 19,978)^e^	
**Comparison of group 2 vs group 3**							
	3-month follow-up	581 (3747 to 4888)		0.002 (–0.002 to 0.006)		—	
	6-month follow-up	4339 (940 to 7738)		0.004 (–0.004 to 0.012)		—	
	9-month follow-up	466 (–2853 to 3784)		–0.006 (–0.014 to 0.002)		—	
	12-month follow-up	2423 (–845 to 5691)		–0.003 (–0.012 to 0.007)		—	
	Total 3 to 12 months	7798 (–2303 to 17,900)		–0.004 (–0.028 to 0.021)		Dominated (dominated to 21,287)	

^a^QALY: quality-adjusted life year.

^b^ICER: incremental cost-effectiveness ratio.

^c^Incremental cost ratio not calculated.

^d^The lower CI is a result of lower costs and fewer incremental quality-adjusted life years.

^e^The bootstrap results are spread across all 4 quadrants of the cost-effectiveness plane, making the CI difficult to interpret.

### Sensitivity Analyses

The results for the comparison of group 2 with the control group were generally robust in the sensitivity analyses as shown in Table 5. The exception was multiple imputation that led to a nonsignificant negative mean difference in QALYs between group 2 and group 1 (control). The probability that the group 2 intervention would be cost-effective compared with the control group at the willingness-to-pay threshold of Aus $50,000 per QALY in the complete case analysis was estimated at 63% (Figure S2 in Multimedia Appendix 1).

The intervention cost of group 2 was also varied to assess the threshold when the mean cost difference between group 2 and group 1 would become positive. This occurred when the group 2 intervention cost was Aus $4500.

The sensitivity analyses for the comparison of group 3 with group 1 (control) were mostly consistent with the base case (Table 5). The analysis using multiple imputation led to negative incremental cost and QALY differences, both being nonsignificant, but leading to a positive incremental ICER. Across all sensitivity analyses, the probability of group 3 being cost-effective compared with group 1 (control) at the threshold of Aus $50,000 per QALY was ≤54% (Figure S3 in Multimedia Appendix 1).

The sensitivity analyses for the comparison of group 2 and group 3 were mixed. The complete case and multiple imputation analyses led to positive mean differences in QALYs and positive ICERs. The probability of group 3 being cost-effective compared with group 2 at the threshold of Aus $50,000 per QALY was ≤22% across all sensitivity analyses (Figure S4 in Multimedia Appendix 1).

Using the costs of the group 2 and group 3 interventions, if implemented across the population of people with BD in Australia (Table S7 in Multimedia Appendix 1), led to marginally lower mean differences in costs, which did not substantially change the ICERs.

**Table 5:** Sensitivity analyses, in Australian dollars (Aus $1=US $0.7058), on incremental cost-utility ratios by randomized group.

		Health care costs, mean difference (95% CI)	QALY^a^, mean difference (95% CI)	Cost per QALY, ICER^b^ (95% CI)
**Comparison of group 2 vs group 1 (control)**				
	Mixed effects model (base case)	–2858 (–10,909 to 5194)	0.012 (–0.009 to 0.033)	Dominant (43,000 to dominant)^c^
	Complete case	–6164 (–12,435 to 108)	0.000 (–0.035 to 0.035)	Dominant (43,000 to dominant)^c^
	Multiple imputation	–2277 (–6568 to 2023)	–0.001 (–0.023 to 0.021)	2,277,000 (19,465 to dominant)
	Population-level intervention costs	–3081 (–11,132 to 4970)	0.012 (–0.009 to 0.033)	Dominant (33,370 to dominant)^c^
**Comparison of group 3 vs group 1 (control)**				
	Mixed effects model (base case)	–94 (–9422 to 9235)	0.002 (–0.023 to 0.027)	Dominant (dominated to 19,978)^d^
	Complete case	–2826 (–10,168 to 4516)	0.005 (–0.032 to 0.042)	Dominant (dominated to 19,978)^c^^d^
	Multiple imputation	–831 (–6943 to 5808)	–0.003 (–0.027 to –0.022)	257,361 (dominated to 35,982)
	Population-level intervention costs	–386 (–9714 to 8943)	0.002 (–0.023 to 0.027)	Dominant (dominated to 18,559)^c^^d^
**Comparison of group 2 vs group 3**				
	Mixed effects model (base case)	7798 (–2303 to 17,900)	–0.004 (–0.028 to 0.021)	Dominated (dominated to 21,287)
	Complete case	3338 (–2072 to 8748)	0.005 (–0.033 to 0.043)	667,600 (dominated to 21,287)
	Multiple imputation	2527 (–3415 to 8469)	0.002 (–0.022 to 0.026)	1,263,500 (dominant to 14,129)
	Population-level intervention costs	7729 (–2372 to 17,831)	–0.004 (–0.028 to 0.021)	Dominated (dominant to 16,283)

^a^QALY: quality-adjusted life year.

^b^ICER: incremental cost-effectiveness ratio.

^c^Complete case bootstrap CIs were used for both mixed effects model and complete case analyses.

^d^The results are spread across all 4 quadrants of the cost-effectiveness plane, making the CI difficult to interpret.

## Discussion

### Principal Findings

To our knowledge, this is the first economic evaluation of an internet-based psychoeducation and CBT intervention specific to people with a diagnosis of BD [18,41]. The results suggest that the psychoeducation offered to group 2 through the MoodSwings 2.0 website may be cost-effective compared with an active control group of a moderated internet-based discussion board for people with a diagnosis of BD. The results also suggest that the addition of CBT tools to the psychoeducation component was not cost-effective compared with the moderated internet-based discussion board alone or the combination of psychoeducation plus the moderated internet-based discussion board.

The difference in cost between the participants randomized to the internet-based psychoeducation and the control condition (internet-based forum only), although not significantly different, showed a trend favoring internet-based psychoeducation. This was attributed to lower costs for acute care services such as hospitalizations and emergency room visits. These results are similar to those of research evaluating the costs and outcomes associated with an in-person 21-session group psychoeducation program for people with BD [42]. Over 5 years of follow-up, participants receiving the group psychoeducation had significantly fewer days hospitalized, which led to nonsignificant lower total costs for the psychoeducation group. Our results contrast with another trial-based cost-effectiveness analysis of an in-person 21-session structured group psychoeducation program that found significantly higher total costs and additional QALY gains for the participants receiving group psychoeducation compared with those receiving unstructured group peer support [43].

The MoodSwings 2.0 study group randomized to the psychoeducation modules showed statistically significant improvements in depression symptoms, as measured by MADRS scores, compared with the control group. These differences were also clinically meaningful, falling within the range of estimated minimal important difference of 3 to 6 points for the MADRS [44]. This is similar to results from the study by Lam et al [45] that found significantly improved scores on the Beck Depression Inventory at 4 and 6 months for the group receiving cognitive therapy versus a control group. The resulting average ICER for the psychoeducation intervention compared with the control group was dominant, meaning that there was improvement in MADRS scores at a cost savings.

Cost-effectiveness ratios such as cost per point change in MADRS score are difficult to interpret because of a lack of value attached to a point change in MADRS score. QALYs have inherent value-for-money connotations because of generally accepted willingness-to-pay thresholds used by health technology assessment agencies such as the United Kingdom’s National Institute for Health and Care Excellence and Australia’s Medicare Services Advisory Committee.

We did not find significant differences in utility values or QALYs among the groups over the 12-month follow-up. This contrasts with a small significant QALY gain of 0.023 (95% CI 0.001-0.56) associated with a previously evaluated in-person group psychoeducation intervention compared with in-person group peer support [43]. This may be due to the in-person mode of delivery, a longer follow-up of 96 months, use of the EQ-5D instrument, and lower rates of loss to follow-up.

Our results further suggest that the combination of psychoeducation and CBT tools (group 3) would not be considered cost-effective compared with the moderated internet-based discussion board (group 1) based on the cost-utility results. Group 3 had a trend toward lower costs and more QALYs compared with the control group, but there was a great deal of uncertainty around this dominant average cost per QALY ratio, resulting in a 51% probability of being cost-effective at the threshold of Aus $50,000 per QALY generally accepted as value for money in Australia. These results are comparable to economic evaluations of other unguided internet-based CBT interventions evaluated in people with unipolar depression [46,47].

The combination of internet-based psychoeducation and CBT tools (group 3) would not be considered good value for money compared with internet-based psychoeducation (group 2) based on the dominated average cost-utility ratio. The combination of internet-based psychoeducation and CBT tools (group 3) resulted in an average cost-effectiveness ratio of Aus $11,140 per point improvement in MADRS compared with internet-based psychoeducation (group 2). Although this seems favorable, it is harder to interpret because we do not have a willingness-to-pay threshold for a point improvement in depression symptom scores.

A prior evaluation of the MoodSwings 2.0 program found within-group improvements in depression and mania symptoms, medication adherence, and quality of life for participants receiving psychoeducation alone and psychoeducation plus CBT-based interactive elements [26]. The lack of an attention control group may explain the difference in findings compared with our evaluation.

### Limitations

As with all research, the results of this economic evaluation are subject to limitations. There was a high rate of loss to follow-up over the 12 months of the study period and a higher likelihood of missing cost and utility data for female participants, which may affect the validity of the results. The cost-effectiveness and cost-utility analyses would only be generalizable to the Australian context because of the exclusive use of Australian unit costs. The analytic approach followed published recommendations for the management of missing data. However, the complete case and multiple imputation results differed from the base case for the comparison of group 2 with group 3 as well as multiple imputation results differing from the base case for the comparison of group 3 with group 1 (control). There was also no treatment-as-usual control arm. The cost of programming and delivering the internet-based interventions was estimated based on the available information for this trial but may have been higher because of additional time for programming and system maintenance not captured in our projected costs. However, we found that the average total cost was lower for group 2 than for group 1 (control) until the intervention cost reached Aus $4500 per study participant, which is 7 times higher than the Aus $645 base case intervention cost for group 2.

Despite this evaluation’s limitations, it is important to report the results of economic evaluations of internet interventions aimed at supporting people with BD. Overall, there is limited literature on the cost-effectiveness of psychosocial interventions for the treatment of BD and none for BD-specific digital interventions [18,31]. People with BD are a unique population because of the symptomatology, medications, and behavioral aspects related to the diagnosis. Psychosocial interventions designed for other mental health conditions (ie, unipolar depression and anxiety) may not be appropriate to extrapolate to people with BD. It is important to tailor the information to the specific issues related to this diagnosis and evaluate program effectiveness and cost-effectiveness.

The availability of internet-based interventions is crucial, given lack of access to mental health professionals because of limited availability, geographic location, and, recently, public health measures related to COVID-19 infection control. The Australian Productivity Commission’s Inquiry Into Mental Health report recommended a national digital mental health platform with a gateway to digital and face-to-face treatment and support services. Any interventions provided through this mental health gateway should have evidence of, or at a minimum be concurrently evaluated for, their effectiveness and cost-effectiveness.

### Conclusions

The internet-based psychoeducation provided through the MoodSwings 2.0 platform to the group 2 participants has the potential to be a cost-effective intervention for people diagnosed with BD. The group 2 psychoeducation component could be further evaluated in an implementation study for effectiveness and cost-effectiveness. Additional research is needed to understand the lack of effectiveness for the internet-based CBT tools provided as part of the group 3 intervention.

## Appendix

Multimedia Appendix 1 Supplementary materials.

## Abbreviations

BD: bipolar disorder
CBT: cognitive behavioral therapy
GLM: generalized linear model
ICER: incremental cost-effectiveness ratio
MADRS: Montgomery-Åsberg Depression Rating Scale
QALY: quality-adjusted life year
RCT: randomized controlled trial
SF-12: short-form health survey, 12-item version
SF-6D: short-form 6-dimension
