# Supplementary material for: Cost-Utility and Cost-effectiveness of MoodSwings 2.0, an Internet-Based Self-management Program for Bipolar Disorder: Economic Evaluation Alongside a Randomized Controlled Trial
Source: JMIR Ment Health. 2022 Nov 1;9(11):e36496. doi: 10.2196/36496 (PMC9667380; doi:10.2196/36496)
Supplement: Multimedia Appendix 1 [file mental_v9i11e36496_app1.docx]

**Multimedia Appendix 1**

**Online Supplementary Materials**

**Table S1. Impact Inventory as recommended by the Second Panel on Cost-Effectiveness in Health and Medicine**

| Sector | Type of Impact | Included in this reference case analysis from …perspective? | | Notes on sources of evidence |
| --- | --- | --- | --- | --- |
|  |  | Health Care Sector | Societal |  |
| Formal Health Care Sector | | | | |
| Health | Health outcomes (effects) | | | |
|  | Longevity effects |  |  |  |
|  | Health -related quality-of-life effects | ✓ |  | SF-6D |
|  | Other health effects (eg, adverse events and secondary transmissions of infections) |  |  |  |
|  | Medical costs | | | |
|  | Paid for by third-party payers | ✓ |  | Self-reported |
|  | Paid for by patients out-of-pocket |  |  |  |
|  | Future related medical costs (payers and patients) |  |  |  |
|  | Future unrelated medical costs (payers and patients) |  |  |  |
| Informal Health Care Sector | | | | |
| Health | Patient-time costs | NA |  |  |
|  | Unpaid caregiver-time costs | NA |  |  |
|  | Transportation costs | NA |  |  |
| Non-Health Care Sectors (with examples of possible items) | | | | |
| Productivity | Labour market earnings lost | NA |  |  |
|  | Cost of unpaid lost productivity due to illness | NA |  |  |
|  | cost of uncompensated household production | NA |  |  |
| Consumption | Future consumption unrelated to health | NA |  |  |
| Social Services | Cost of social services as part of intervention | NA |  |  |
| Legal or criminal justice | Number of crimes related to intervention | NA |  |  |
|  | Cost of crimes related to intervention | NA |  |  |
| Education | Impact of intervention on educational achievement of population | NA |  |  |
| Housing | Cost of intervention on home improvements (eg, removing lead paint) | NA |  |  |
| Environment | production of toxic waste pollution by intervention | NA |  |  |
| Other (specify) |  | NA |  |  |

Template based on Figure 1 from Sanders et al, JAMA 2016.

**Figure S1. CONSORT diagram for patient flow** **as reported in Gliddon et al., 2019.[23]**


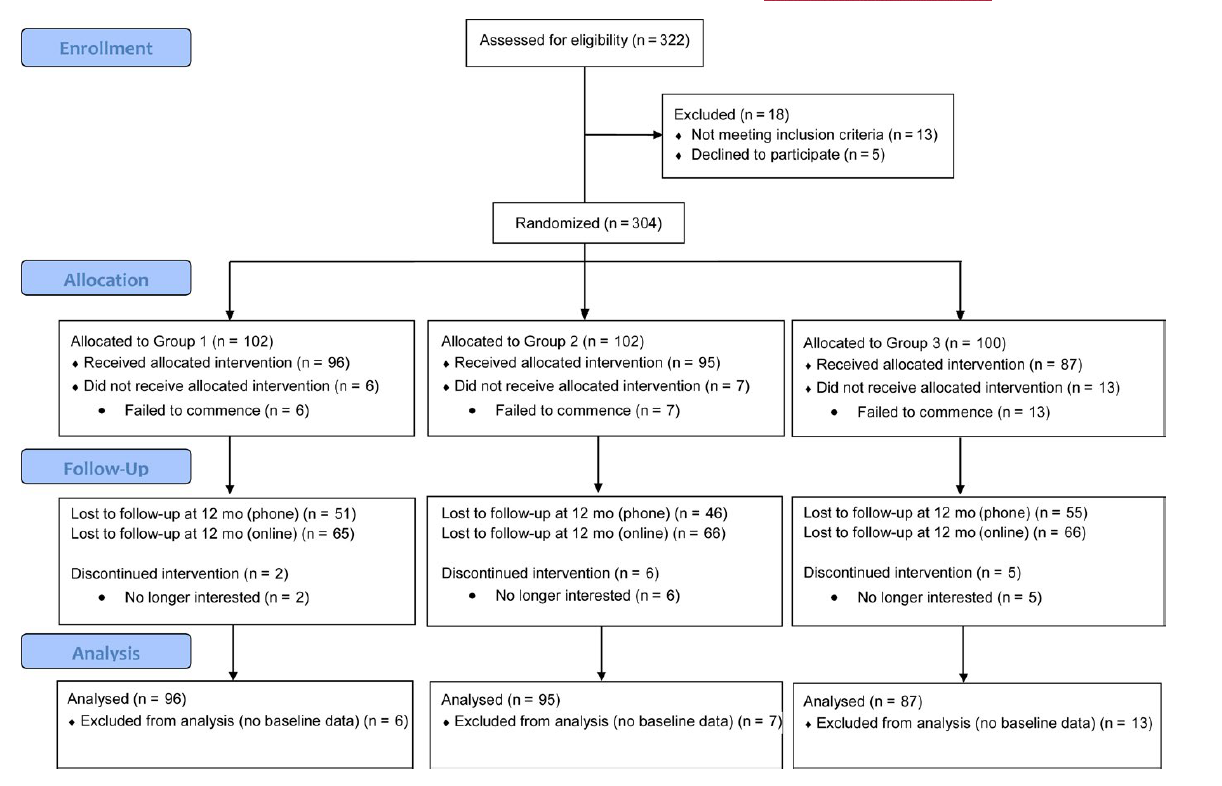


**Table S2. Number and percent of participants with complete cost and utility data by follow-up period and randomised group**

|  | **Group 1 (control)** | **Group 2** | **Group 3** | **Overall sample** |
| --- | --- | --- | --- | --- |
|  | **(N=102)** | **(N=102)** | **(N=100)** | **(N=304)** |
|  | **N (%)** | **N (%)** | **N (%)** | **N (%)** |
| Baseline | 96 (94) | 95 (93) | 87 (87) | 278 (91) |
| 3-month follow-up | 47 (46) | 35 (34) | 38 (38) | 120 (39) |
| 6-month follow-up | 42 (41) | 31 (30) | 30 (30) | 103 (34) |
| 9-month follow-up | 41 (40) | 37 (36) | 30 (30) | 108 (36) |
| 12-month follow-up | 33 (32) | 30 (29) | 26 (26) | 89 (29) |
| Overall | 33 (32) | 29 (28) | 22 (22) | 84 (28) |

**Table S3. Health sector cost by follow-up period and randomised group**

|  | **Group 1 (control)** | **Group 2** | **Group 3** | **1 vs. 2** | **1 vs. 3** | **2 vs.3** |
| --- | --- | --- | --- | --- | --- | --- |
|  | **Mean (SD)** | **Mean (SD)** | **Mean (SD)** | ***P* value^b^** | ***P* value^b^** | ***P* value^b^** |
| Baseline | $4,192 ($7,731) | $3,472 ($6,952) | $2,957 ($5,793) | 0.69 | 0.08 | 0.15 |
| 3-month follow-up^a^ | $3,774 ($4,500) | $3,121 ($4,522) | $4,720 ($14,818) | 0.98 | 0.96 | 0.80 |
| 6-month follow-up | $3,502 ($10,590) | $1,666 ($1,707) | $4,208 ($10,137) | 0.46 | 0.87 | 0.01 |
| 9-month follow-up | $3,445 ($7,277) | $3,365 ($7,318) | $3,548 ($8,070) | 0.65 | 0.73 | 0.78 |
| 12-month follow-up | $3,529 ($9,496) | $2,128 ($2,913) | $2,877 ($10,212) | 0.65 | 0.73 | 0.15 |
| Total | $15,175 ($17,206) | $9,431 ($8,540) | $15,518 ($30,523) | 0.49 | 0.98 | 0.13 |

^a^Health sector cost at 3 months included intervention cost as well as additional health care services. ^b^The analysis of baseline cost data used a generalised linear model with gamma family and identity link. The analysis of data at the remaining follow-ups was analysed using a linear mixed model.

**Table S4. Group means by cost categories across 3–12-month follow-up**

|  | **Group 1 (control)** | | **Group 2** | | **Group 3** | |
| --- | --- | --- | --- | --- | --- | --- |
|  | **(N=33)** | | **(N=30)** | | **(N=22)** | |
|  | **Mean** | **SD** | **Mean** | **SD** | **Mean** | **SD** |
| Intervention | $421 |  | $645 |  | $714 |  |
| Medical services | $2,916 | $1,978 | $3,228 | $2,215 | $3,505 | $4,281 |
| Psychological services | $5,742 | $6,487 | $4,398 | $6,489 | $3,657 | $4,688 |
| Professional support | $56 | $165 | $175 | $790 | $1,182 | $4,805 |
| Acute care | $6,040 | $15,152 | $1,015 | $2,206 | $6,461 | $19,516 |
| Total cost | $15,175 | $17,206 | $9,431 | $8,540 | $15,518 | $30,523 |

**Table S5. Montgomery-Asberg Depression Rating Scale (MADRS) scores by follow-up period and randomised group as reported in Gliddon et al., 2019. [23]**

|  | **Group 1 (Control)** | **Group 2** | **Group 3** | **1 vs. 2** | **1 vs. 3** | **2 vs.3** |
| --- | --- | --- | --- | --- | --- | --- |
|  | **Mean (SD)** | **Mean (SD)** | **Mean (SD)** | ***P* value** | ***P* value** | ***P* value** |
| Baseline | 9.0 (8.5) | 11.2 (8.7) | 9.7 (8.4) |  |  |  |
| 3-month follow-up | 12.0 (9.2) | 9.9 (7.7) | 11.6 (10.2) | 0.04 | 0.60 | 0.45 |
| 6-month follow-up | 12.4 (9.3) | 8.7 (7.5) | 7.8 (7.2) | <.01 | 0.01 | 0.64 |
| 9-month follow-up | 11.8 (7.2) | 10.1 (8.4) | 9.7 (8.3) | 0.05 | 0.19 | 0.89 |
| 12-month follow-up | 11.6 (9.5) | 10.1 (8.4) | 8.6 (9.3) | 0.05 | 0.08 | 0.64 |

**Table S6. Utility values and quality adjusted life years (QALYs) by follow-up period and randomised group**

|  | **Group 1 (control)** | **Group 2** | **Group 3** | **1 vs. 2** | **1 vs. 3** | **2 vs. 3** |
| --- | --- | --- | --- | --- | --- | --- |
|  | **Mean (SD)** | **Mean (SD)** | **Mean (SD)** | ***P* value^b^** | ***P* value^b^** | ***P* value^b^** |
| Baseline^a^ | 0.639 (0.086) | 0.633 (0.085) | 0.629 (0.073) | 0.58 | 0.39 | 0.74 |
| 3-month follow-up | 0.628 (0.098) | 0.634 (0.096) | 0.633 (0.126) | 0.82 | 0.76 | 0.37 |
| 6-month follow-up | 0.627 (0.101) | 0.613 (0.090) | 0.610 (0.093) | 0.32 | 0.66 | 0.36 |
| 9-month follow-up | 0.619 (0.080) | 0.642 (0.092) | 0.623 (0.092) | 0.26 | 0.90 | 0.12 |
| 12-month follow-up | 0.651 (0.120) | 0.637 (0.127) | 0.636 (0.109) | 0.33 | 0.95 | 0.58 |
| QALYs | 0.627 (0.062) | 0.618 (0.094) | 0.622 (0.087) | 0.25 | 0.89 | 0.78 |

^a^The analysis of baseline utility data used an ordinary least squares regression model. ^b^The analysis of data at the remaining follow-ups was analysed using a linear mixed model.

**Table S7. Estimated cost for roll out of Group 2 and 3 interventions**

| **Item** | **Total cost** | **Group 2 Forum + psychoeducation** | **Group 3 Forum+ psychoeducation+ CBT tools** | **Estimated number of people with BD seeking care** | **Average cost per person with BD** |
| --- | --- | --- | --- | --- | --- |
| **Fixed cost** |  |  |  |  |  |
| Website development and maintenance | $120,000.00 |  |  | 108,991 | $1.10 |
| **Variable cost** |  |  |  |  |  |
| Desktop computers | $3,590.14 | $1,204.59 | $1,180.97 |  |  |
| Personnel |  |  |  |  |  |
| Research assistant time to monitor online forum | $44,052.46 | $14,778.81 | $14,490.94 |  |  |
| Research assistant time for debriefing | $4,393.04 | $1,477.88 | $1,445.08 |  |  |
| Supervisor time for debriefing | $8,015.85 | $2,689.53 | $2,636.79 |  |  |
| Total variable cost | $60,051.48 | $20,150.80 | $19,753.78 |  |  |
| Average variable cost per person with BD |  | $197.56 | $197.54 |  |  |
| **Estimated cost with population level roll out** |  | $198.66 | $198.64 |  |  |

**Figure S2. Acceptability curve for cost-utility analysis of Group 2 versus Group 1 constructed from complete case bootstrapped iterations**

**Figure S3. Acceptability curve for cost-utility analysis of Group 3 versus Group 1 constructed from complete case bootstrapped iterations**

**Figure S4. Acceptability curve for cost-utility analysis of Group 3 versus Group 2** **constructed from complete case bootstrapped iterations**
